# Screening of Mercury Bioaccumulation in Birds (Passeriformes) from a Major Wetland of the Brazilian Cerrado

**DOI:** 10.1007/s00128-026-04186-2

**Published:** 2026-02-09

**Authors:** Lucas Cabrera Monteiro, Renato Caparroz, Marcelo Lisita Junqueira, Pedro Henrique Vogeley, Ludgero Cardoso Galli Vieira, José Vicente Elias Bernardi

**Affiliations:** 1 Programa de Pós-Graduação em Ecologia, Departamento de Ecologia, Instituto de Ciências Biológicas, Universidade de Brasília, Brasília, DF Brazil; 2 Núcleo de Estudos e Pesquisas Ambientais e Limnológicas, Faculdade UnB Planaltina, Universidade de Brasília, Brasília, DF Brazil; 3 Laboratório de Geoestatística e Geodésia, Faculdade UnB Planaltina, Universidade de Brasília, Brasília, DF Brazil; 4 Laboratório de Genética e Biodiversidade, Departamento de Genética e Morfologia, Instituto de Ciências Biológicas, Universidade de Brasília, Brasília, DF Brazil; 5 Laboratório de Etnobiologia e Biodiversidade, Universidade Federal de Goiás, Goiânia, Goiás, Brazil; 6 Programa de Pós-Graduação em Ciências Ambientais, Faculdade UnB Planaltina, Universidade de Brasília, Brasília, DF Brazil

**Keywords:** Biomarkers, Bioaccumulation, Trophic biomagnification, Riparian ecosystems, Wetland, Araguaia River

## Abstract

We quantified total mercury (THg) in feathers of Passeriformes from the Araguaia River floodplain (Brazil) to assess bioaccumulation and biomagnification across trophic guilds. Thirty-nine individuals from 22 non-migratory species were analyzed. Feather THg ranged from 0.05 to 2.35 mg kg^−1^ (d.w.), with highest concentrations in invertivores. Invertivorous birds exhibited consistently high biomagnification factors for all invertebrate prey. Omnivorous birds showed lower and more variable biomagnification, suggesting dietary diversification attenuates Hg transfer. Herbivorous birds also showed significant biomagnification, highlighting basal compartments as relevant exposure pathways. Feathers proved to be effective non-lethal indicators of Hg dynamics in Neotropical riparian food webs.

**Supplementary Information:**

The online version contains supplementary material available at 10.1007/s00128-026-04186-2.

## Introduction

Mercury (Hg) is a priority pollutant due to its high mobility, bioaccumulation, and biomagnification through trophic webs, posing risks to ecosystems and human health (UNEP 2019). In Neotropical environments distant from point sources, land-use changes enhance Hg transport to aquatic ecosystems (Fisher et al. 2023). Under anaerobic conditions, inorganic Hg is converted to methylmercury (MeHg), facilitating its incorporation into aquatic food webs and subsequent transfer to terrestrial systems via trophic pathways (Jackson et al. 2021). By exploiting resources derived from both aquatic and terrestrial compartments and serving as prey for higher-level predators, small birds integrate Hg fluxes across the aquatic–terrestrial interface (Barnes and Gerstenberger 2015). Accordingly, many studies have used bird feathers as biomarkers of Hg distribution, including for the identification of emission sources and the assessment of its geographic and trophic distribution in the environment (Sayers et al. 2023; Pisconte et al. 2024; de Paula et al. 2025).

A recent systematic review indicated that, among 22 studies assessing Hg concentrations in birds in Brazil, only one focused on species of the order Passeriformes (de Paula et al. 2025). This single study, conducted by França et al. (2010), was primarily dedicated to validating an analytical method for trace element determination and did not evaluate the influence of trophic level. Passeriformes represent the most diverse order among birds, with a wide geographic distribution and a broad range of trophic strategies (Pigot et al. 2016; Oliveros et al. 2019), making them ideal models for assessing contaminant bioaccumulation in terrestrial ecosystems. Despite their ecological importance, there are no available data on Hg bioaccumulation in birds from the Cerrado biome (Brazilian savanna), which is recognized as a biodiversity hotspot due to its high diversity of endemic species and rapid habitat degradation.

We collected birds of the order Passeriformes inhabiting riparian ecosystems of the Araguaia River floodplain to quantify total mercury (THg) concentrations, compare bioaccumulation among trophic guilds, and estimate biomagnification based on previously reported Hg concentrations in plant resources and invertebrates from the floodplain (Monteiro et al. 2024). This screening provides novel data for the Cerrado biome and contributes to expanding the information on bioaccumulation in Passeriformes species in Neotropical environments.

## Material and Methods

Our study was conducted in the Araguaia River floodplain, in the municipality of Aruanã, Goiás State (Central-West Brazil). Birds were collected in November 2023 from a semi-deciduous forest area located approximately 700 m from the Araguaia River (S 14° 51′ 01,4″; W 51**°** 04′ 53,2). Birds were captured using 12 × 3 m nylon mist nets, which were opened for approximately three hours after sunrise and two hours before sunset. Captured individuals were kept in cotton bags in a shaded area until feather collection. Five to seven dorsal feathers were collected from each bird and stored using plastic bags at ambient temperature. After sampling, all birds were released at their capture sites.

A total of 39 individuals from 22 species of the order Passeriformes were collected. The species were classified into three trophic guilds, according to Wilman et al. (2014): herbivore (n = 7), omnivore (n = 10), and invertivore (n = 22) (Table S1). No migratory species were collected; therefore, feather Hg concentrations are interpreted as reflecting local exposure to Hg sources within the Araguaia River basin. Feather samples were immersed in acetone (≥ 99.9%, HPLC grade, Sigma-Aldrich, St. Louis, MO, USA) for 30 min, rinsed three times with deionized water, and oven-dried at 50 °C for 48 h (Ma et al. 2021). The dried samples were then finely cut using pre-heated stainless-steel scissors, which were cleaned with absolute ethanol (99.5%, ACS grade, Dinâmica Química, São Paulo, Brazil) between samples to prevent cross-contamination.

Total Hg concentrations were determined by thermal desorption atomic absorption spectrometry using a RA-915 + analyzer coupled with a pyrolysis chamber (Pyro-915 +) (Lumex, St. Petersburg, Russia). The limit of detection (LOD) was 0.0001 mg kg^−1^ (d.w.). The analytical blank was obtained by measuring THg in empty quartz boats for 45 s, yielding absorbance signals consistently below the detection limit. Duplicates were analyzed every ten samples, showing relative percent differences below 10% (n = 6). Analytical accuracy was assessed using certified reference materials DORM-2 (Dogfish muscle, National Research Council, Canada) and IAEA-142 (Mussel homogenate, International Atomic Energy Agency, Austria), with mean recovery rates of 97.8 ± 4.2% and 92.8 ± 4.3%, respectively (n = 4 each).

Total Hg concentrations were reported as mean ± standard deviation (d.w.). Differences in THg concentrations among trophic guilds were assessed using the Kruskal–Wallis test followed by Dunn’s post hoc test. We used data on THg concentrations in leaf litter, fresh leaves, and invertebrates from the Araguaia River floodplain published by Monteiro et al. (2024) to evaluate the degree of biomagnification in birds. The biomagnification factor (BMF) was calculated as the ratio between THg concentrations in predators and their food source (Conder et al. 2012), using a nonparametric bootstrap procedure with 10,000 iterations. A directed trophic network was subsequently constructed, with nodes representing taxa or environmental compartments and edges representing trophic interactions weighted by the median BMF. Concentrations below the LOD were not included in the statistical analyses (n = 2). All statistical analyses were conducted in the R environment (R Core Team, 2025), with the significance level set at p < 0.05.

## Results and Discussion

**Fig. 1:**
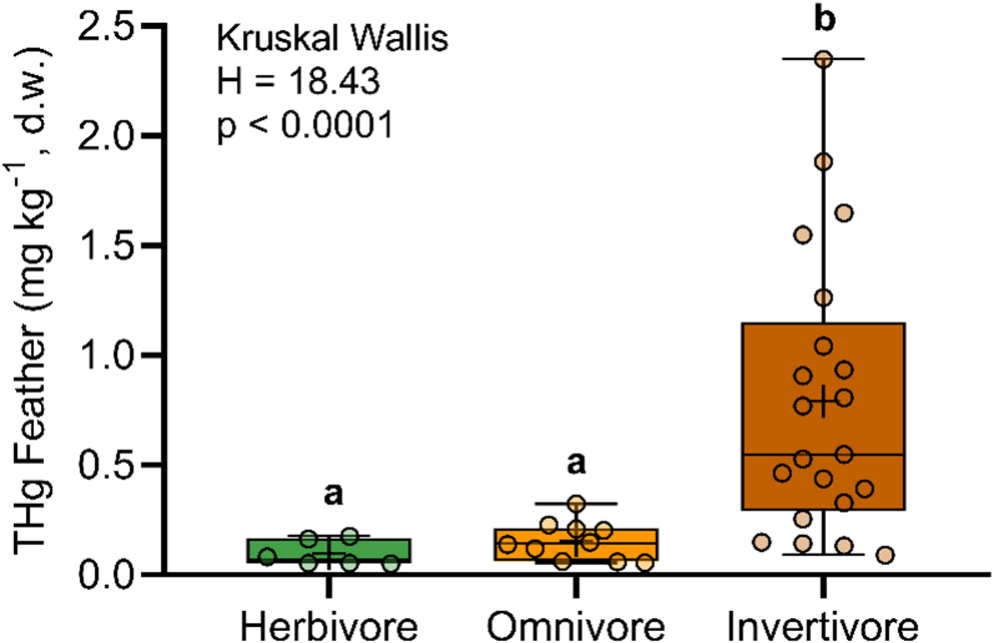
Comparison of THg concentrations among trophic guilds. The letters “a” and “b” indicate significant differences determined by Dunn's post hoc test (p < 0.05)

Total Hg concentrations in feathers ranged from 0.05 to 2.35 mg kg^−1^, with a mean of 0.51 ± 0.58 mg kg^−1^ (Table S1). THg concentrations in invertivorous species (0.79 ± 0.63 mg kg^−1^, n = 21) were significantly higher than those determined in herbivorous (0.10 ± 0.06 mg kg^−1^; n = 6; p = 0.0008) and omnivorous species (0.15 ± 0.09 mg kg^−1^; n = 10; p = 0.004) (H = 18.43; p < 0.0001) (Fig. 1), consistent with feather THg concentrations found across similar trophic distributions of avian foraging guilds by others studies (Barnes and Gerstenberger 2015; Sayers et al. 2023; Pisconte et al. 2024). Because passerines often serve as prey for higher trophic levels, this pattern may enhance Hg biomagnification, as invertivorous passerines exhibit higher Hg concentrations than other dietary guilds (Barnes and Gerstenberger 2015).

**Fig. 2:**
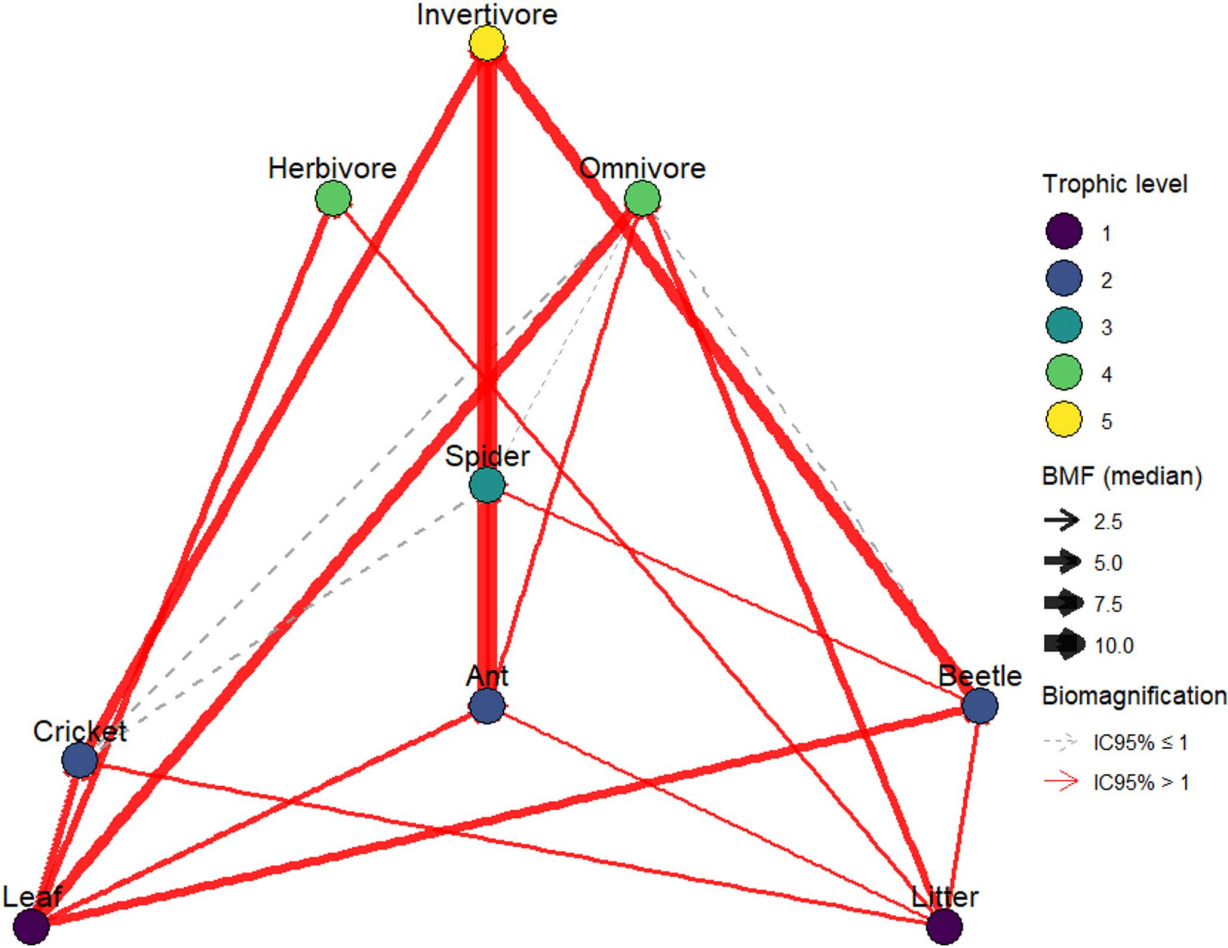
Trophic network showing Hg biomagnification pathways among basal resources, invertebrates, and avian trophic guilds. Data on THg in basal resources and invertebrates were obtained from Monteiro et al. (2024). Node colors indicate trophic level. Directed edges represent prey–predator relationships; edge width is proportional to the median biomagnification factor (BMF) estimated by bootstrap (10,000 iterations). Solid red arrows indicate links with IC95% > 1, while dashed gray arrows indicate links with IC95% ≤ 1

The biomagnification factors (BMFs) based on plant resources and invertivores data from Monteiro et al. (2024) confirmed marked differences among avian trophic guilds (Fig. 2). Invertivorous birds exhibited consistently high BMFs for all invertebrate prey. Omnivorous birds showed lower and more variable BMFs, with significant biomagnification detected only for ants and plant resources, suggesting that dietary diversification attenuates Hg transfer. Herbivorous birds also showed BMFs > 1 for leaves and litter, indicating basal compartments as relevant exposure pathways. Moreover, the proximity to aquatic ecosystems may enhance the predation of emerging aquatic invertebrates, which exhibit high proportions of MeHg, thereby intensifying Hg bioaccumulation in birds (Jackson et al. 2021).

Feather THg concentrations determined in our study were substantially lower than those reported for other regions of Brazil. Most of those studies focused on piscivorous species and/or birds with considerably larger body sizes than those analyzed here (de Paula et al. 2025). Only França et al. (2010) evaluated Hg concentrations in individuals of the order Passeriformes, specifically in the species *Arremon flavirostris*, *Myiothlypis flaveola*, and *Synallaxis spixi*, with concentrations ranging from < 0.006 to 0.3 mg kg^−1^. In the Peruvian Amazon, Hg concentrations in *Ramphocelus carbo* were 0.20 mg kg^−1^ in a natural area (Pisconte et al. 2024), within the range observed in our study (0.06—0.23 mg kg^−1^). Pisconte et al. (2024) reported concentrations between 0.13 and 1.25 mg kg^−1^ in *Inezia inornata* under ASGM influence, whereas we found 1.04 mg kg^−1^ in a species of the same genus (*Inezia subflava*). These findings demonstrate that, even in the absence of significant ASGM Hg sources, dietary exposure is still an important driver of bioaccumulation.

The marked THg differences we observed between trophic guilds highlights the usefulness of dorsal feathers as a non-lethal biomarker for assessing Hg exposure, bioaccumulation, and with sufficient associated environmental occurrence data, availability and biomagnification in birds. Our study represents a preliminary screening of Hg occurrence in Passeriformes species from the Cerrado biome. Further studies with larger sample sizes are recommended, including Hg quantification and speciation in potential dietary sources such as seeds, fruits, invertebrates, and even incidental soil or sediments (Beyer et al. 1994), all occurring and collected in co-located areas in consistent and relevant times of the year with birds assessed for feather THg.

## Supplementary Information

Below is the link to the electronic supplementary material.

Supplementary Material 1

## Data Availability

Data will be made available on request.
