# Supplementary material for: Screening of Mercury Bioaccumulation in Birds (Passeriformes) from a Major Wetland of the Brazilian Cerrado
Source: Bull Environ Contam Toxicol. 2026 Feb 9;116(2):36. doi: 10.1007/s00128-026-04186-2 (PMC12886310; doi:10.1007/s00128-026-04186-2)
Supplement: Supplementary file 1 — Supplementary Material 1 [file 128_2026_4186_MOESM1_ESM.docx]

**Supplementary Material**

**Table S1.** Mean ± standard deviation and range of total mercury (THg) concentrations in feathers. “Fruit/Nectar” and “Plant/Seed” indicate the food preferences of herbivorous species (Wilman et al., 2014).

|  | **Species** | **Family** | **Feeding habit** | **N** | **THg (mg kg^-1^)** |
| --- | --- | --- | --- | --- | --- |
|  | *Euphonia chlorotica* | Fringillidae | Herbivore_Fruit/Nectar_ | 1 | 0.05 |
|  | *Pipra fasciicauda* | Pipridae | Herbivore_Fruit/Nectar_ | 4 | 0.09 ± 0.06  (0.05 – 0.18) |
|  | *Sicalis flaveola* | Thraupidae | Herbivore_Plant/Seed_ | 1 | <0.0001 |
|  | *Sporophila leucoptera* | Thraupidae | Herbivore_Plant/Seed_ | 1 | 0.16 |
|  | *Pitangus sulphuratus* | Pipridae | Omnivore | 1 | 0.32 |
|  | *Ramphocelus carbo* | Thraupidae | Omnivore | 8 | 0.15 ± 0.06  (0.06 - 0.23) |
| *Elaenia spectabilis* | | Tyrannidae | Omnivore | 1 | 0.06 |
|  | *Cantorchilus leucotis* | Troglotydae | Invertivore | 1 | 0.91 |
|  | *Pheugopedius genibarbis* | Troglotydae | Invertivore | 2 | 1.06 ± 0.84  (0.46 – 1.65) |
|  | *Certhiaxis sp.* | Furnariidae | Invertivore | 1 | 0.55 |
|  | *Cranioleuca vulpina* | Furnariidae | Invertivore | 1 | 1.26 |
|  | *Eucometis penicillata* | Thraupidae | Invertivore | 1 | 0.39 |
|  | *Tachyphonus rufus* | Thraupidae | Invertivore | 1 | 0.13 |
|  | *Paroaria baeri* | Thraupidae | Invertivore | 2 | 0.67 ± 0.20  (0.53 – 0.81) |
|  | *Cnemotriccus fuscatus* | Tyrannidae | Invertivore | 2 | 0.29 ± 0.21  (0.14 - 0.44) |
|  | *Inezia subflava* | Tyrannidae | Invertivore | 1 | 1.04 |
|  | *Formicivora grisea* | Thamnophilinae | Invertivore | 3 | 0.24 ± 0.09  (0.15 - 0.33) |
|  | *Thamnophilus pelzelni* | Thamnophilidae | Invertivore | 1 | 0.09 |
|  | *Hylophilus pectoralis* | Vireonidae | Invertivore | 2 | 1.33 ± 0.79  (0.77 - 1.88) |
|  | *Poecilotriccus latirostris* | Rhynchocyclidae | Invertivore | 1 | <0.0001 |
|  | *Polioptila dumicola* | Polioptilidae | Invertivore | 1 | 2.35 |
|  | *Xiphorynchus guttatoides* | Dendrocolaptidae | Invertivore | 2 | 1.24 ± 0.43  (0.93 - 1.55) |
